# A Computational Study of Amensalistic Control of *Listeria monocytogenes* by *Lactococcus lactis* under Nutrient Rich Conditions in a Chemostat Setting

**DOI:** 10.3390/foods5030061

**Published:** 2016-09-09

**Authors:** Hassan Khassehkhan, Hermann J. Eberl

**Affiliations:** 1 Faculty of Applied Science and Technology, Sheridan College, 7899 McLaughlin Rd, Brampton, ON, L6Y 5H9, Canada; hassan.khassehkhan@sheridancollege.ca; 2 Department of Mathematics and Statistics, University of Guelph, 50 Stone Rd E, Guelph, ON, N1G 2W1, Canada

**Keywords:** *Listeria monocytogenes*, *Lactococus lactis*, mathematical model, computer simulation, biocontrol, 87.10.Ed, 87.23.Cc

## Abstract

We study a previously introduced mathematical model of amensalistic control of the foodborne pathogen *Listeria monocytogenes* by the generally regarded as safe lactic acid bacteria *Lactococcus lactis* in a chemostat setting under nutrient rich growth conditions. The control agent produces lactic acids and thus affects pH in the environment such that it becomes detrimental to the pathogen while it is much more tolerant to these self-inflicted environmental changes itself. The mathematical model consists of five nonlinear ordinary differential equations for both bacterial species, the concentration of lactic acids, the pH and malate. The model is algebraically too involved to allow a comprehensive, rigorous qualitative analysis. Therefore, we conduct a computational study. Our results imply that depending on the growth characteristics of the medium in which the bacteria are cultured, the pathogen can survive in an intermediate flow regime but will be eradicated for slower flow rates and washed out for higher flow rates.

## 1. Introduction

Safe food is a major contributing factor to human health. The control of foodborne pathogenic bacteria plays an important role in food safety, for example by preservation of foods. Among the traditional methods of food preservation are fermentation, temperature treatment (heat/cold/freezing), or the addition of chemical adjuncts to foods, including spices [1]. A newer concept of food preservation or microbial food safety control could be described as “ecological control”, in which the microbial ecology of the food is augmented by bacteria that are *generally regarded as safe (GRAS)*, with the goal to either out-compete the pathogen, or to change the local environmental conditions such that they become detrimental for the pathogen. Examples for this concept can be found in [1,2,3,4] and the references that they cite. Since this approach is based on the abilities of living microorganisms, it belongs to the so-called biopreservation techniques, in which in particular lactic acid bacteria play an important role [1]. Other biopreservation techniques include control based on microbially produced bacteriocins, such as natural antimicrobials, or so called “hidden fermentation”.

We study here a mathematical model of biopreservation, or biocontrol, of the pathogenic *Listeria monocytogenes* by the lactic acid bacteria *Lactococus lactis*, based on earlier work in [2]. The use of such an ecological control mechanism has been proposed for minimally processed refrigerated vegetable products [1,3].

*L. monocytogenes* is a pathogenic bacteria, which can cause the sometimes fatal disease listeriosis. While individuals with normal health may not develop symptoms, it can be deadly for fetuses, newborns, infants, the elderly, pregnant women, and immuno-compromised people [5]. An outbreak of listeriosis in Canada in 2008 that was traced back to a meat processing facility caused several deaths [6]. *L. monocytogenes* can be found in uncooked meat, milk, egg, seafoods, and fish as well as certain minimally refrigerated vegetables, but also heat-processed foods such as pasteurized milk and dairy products and ready-to-eat meat [5]. In January through May 2016, the Canadian Food Inspection Agency reported at least 20 food recalls and/or alerts because of this pathogen, the majority of which classified as Class I (or *high risk*) [7]. Due to its importance from a food safety perspecitve, mathematical modeling to characterize the growth *L. monocytogenes* has been a very active field of research for more than 20 years. Most studies focus on mono-culture settings, where a wide range of modeling tools is used, from data-driven descriptive fitting, to agent based models, to dynamic differential equations; some such examples are [8,9,10,11,12,13,14,15,16,17], but this list is by no means complete. Fewer models have been proposed to study the dynamics of interaction of *L. monocytogenes* in co-culture with other species [2,18,19,20], which is the setting that is relevant for the ecological control aspect that we focus on.

The control agent *L. lactis* is a lactic acid bacteria that is associated with certain dairy products [1]. While it plays an important role in food microbiology because of its fermentation abilities [5], we are here interested in the changes that it inflicts to the microbial ecology by production of lactic acids and subsequent changes of pH. Simply described, the underlying control mechanism is the following amensalistic principle: the control agent produces lactic acids which decrease the pH in the system. Both, decreasing pH and increasing lactic acid concentration are detrimental to the pathogen, while the control agent itself is much more tolerant to these self-inflicted environmental changes. In the mathematical formulation, this is described by a nonlinear system of five ordinary differential equations [2] for the dependent variables population sizes of pathogen and control agent, concentration of lactic acids, concentration of hydrogen ions (as a measure of pH), and malate. Unlike [2], where the model was studied quantitatively with relatively few computer simulations for a batch culture, we investigate it in a continuous chemostat setup aiming at a qualitative description of the longterm dynamics and how it depends on environmental conditions. The mathematical theory for the chemostat, in which several species compete for a limited number of resources, such as nutrients, is well understood, culminating in a mathematically rigorous formulation of the principle of competitive exclusion [21]. However, if additional effects of interaction between species are at play that can give one competitor an additional growth advantage over the other one, this result cannot be readily applied, because it makes heavy use of a certain functional relationship between microbial growth and substrate depletion that no longer holds. Such systems need to be studied individually, cf. [20,22,23,24]. This is also the situation for the amensalistic control system at hand. Moreover, while the principle of competitive exclusion implies that only one of two competitors for a shared resource in a competition based chemostat can survive, it is known from a variety of examples that pH values and dilution rate can determine the fate of co-cultures in chemostats, and that indeed both competitors can survive, cf. [25] for an experimental study. In our specific example, the pH is actually modified by the species themselves. In our model we implicitly assume that nutrient availability is not limiting bacterial growth. This assumption is made, because the set of reaction parameters that we use in our study was determined in [2,14] from experiments in vegetable broth, where nutrients are plenty. Including nutrient limitation effects and competition for nutrients in the model would be straightforward, but at the expense of introducing additional unknown model parameters and dependent variables, thus increasing the model complexity, and potentially shifting the focus away from amensalistic effects.

The algebraic expressions arising from our model are too involved to allow for a complete and rigorous analytical treatment. Therefore, we study the system with a mix of computational methods and analytical techniques, the latter restricted to relative simple special questions, such as stability of trivial equilibria, or to derive estimates on critical flow rates. In our model, the reaction terms contain in total 18 parameters. A complete set of values for these are given in [2]. With growth kinetics fixed, we will focus on the influence of the reactor operating conditions. These are the flow rate and the properties of the growth medium. In our case these are expressed in terms of the bulk concentrations of lactic acids and hydrogen ions. Thus, we explore the behavior of the system with respect to three parameters.

We end this brief introduction with two remarks. First, lactic acid bacteria play also a major role in fermentation. Fermentation is an important, by some accounts the most important, mechanism for food preservation. This is not addressed by our study, which solely focuses on the control of pathogens; Secondly, the biocontrol mechanism that we study is based on a simple ecological (amensalistic) principle: a (possibly invasive) species alters the environmental conditions such that they become less favorable and detrimental for a competitor, while the control agent itself is more tolerant toward these self-inflicted changes. This gives a natural growth advantage. Lactic acid bacteria are often also used as probiotics, which are defined to be live food ingredients which confer health benefits to the host if administered in sufficient quantities [26]. The same amensalistic control mechanism that we study here in the context of food preservations can also play a role as a probiotic control mechanism. Although the parameter values likely will be different, the model itself might apply to this situation as well.

## 2. Governing Equations

In [2] a mathematical model for the control of *Listeria monocytogenes* by *Lactococus lactis* was formulated for the case of batch cultures. We will study this system in the chemostat setting. It is formulated for the five dependent variables

$N_{1}$: population size of *L. monocytogenes*,
$N_{2}$: population size of *L. lactis*,
*C*: concentration of lactic acids,
*P*: concentration of hydrogen ions,
*M*: malate concentration.

The hydrogen ion concentration is equivalent to the pH value in the sense that

$$\text{pH}=-\log_{10}P,$$

if *P* is measured in moles.

In the chemostat setup, the dissolved substrates malate, lactic acids and hydrogen ions are added to the reactor at a constant reactor flow rate *q*, at bulk concentration levels $C_{0},\;P_{0}$ and $M_{0}$. The bacterial populations, and the dissolved growth limiting substrates are completely mixed and subject to convective transport into and out of the reactor. Thus the reactor is continuously replenished with fresh medium.

The bacterial populations grow if *C* and *P* are small; they decay if one of these concentrations becomes large. Growth and decay phases are separated by extended neutral phases.

Both bacterial species produce lactic acids until a saturation level is reached. Similarly, lactic acids increase the concentration of hydrogen ions (i.e., decrease the pH) until a saturation level is reached. Moreover, hydrogen ions are diminished by *L. lactis*, during which process also malate decays.

This is modeled by the nonlinear system of five ordinary differential equations

(1) $$\begin{array}{ccc} \frac{dN_{1}}{dt} & = & \mu_{1}g_{1}\left(C,P\right)N_{1}-qN_{1}, \end{array}$$

(2) $$\begin{array}{ccc} \frac{dN_{2}}{dt} & = & \mu_{2}g_{2}\left(C,P\right)N_{2}-qN_{2}, \end{array}$$

(3) $$\begin{array}{ccc} \frac{dC}{dt} & = & \gamma N_{1}\left(1-\frac{C}{k_{1}}\right)+\delta N_{2}\left(1-\frac{C}{k_{2}}\right)+q\left(C_{0}-C\right), \end{array}$$

(4) $$\begin{array}{ccc} \frac{dP}{dt} & = & \rho C\left(1-\frac{P}{k_{11}}\right)-\kappa \theta N_{2}M+q\left(P_{0}-P\right), \end{array}$$

(5) $$\begin{array}{ccc} \frac{dM}{dt} & = & -\theta N_{2}M+q\left(M_{0}-M\right). \end{array}$$

In (1) and (2) the constants $\mu_{1}>0,\;\mu_{2}>0$ are the maximum specific growth rates of *L. monocytogenes* and *L. lactis*. In (3), the constants $k_{1}>0$ and $k_{2}>0$ are the saturation levels for lactic acid production and γ>0 and δ>0 the production rates. Similarly in (4), parameter $k_{11}>0$ is the saturation level for production of *P* and ρ>0 the production rate. Constant θ>0 is the rate at which malate is decayed by *L. lactis*, while the rate of decay of *P* in this process is given by κθ>0.

The growth functions $g_{1}\left(C,P\right)$ of *L. monocytogenes* and $g_{2}\left(C,P\right)$ of *L. lactis* are defined by minimal inhibitory concentrations (MIC) for growth and metabolism. In [2], based on the earlier work [14], the following piecewise linear functions are suggested for non-negative arguments

$$g_{1}\left(C,P\right)=\min\left\{1-\frac{C}{H_{1}\left(C\right)},1-\frac{P}{H_{2}\left(P\right)}\right\}$$

and

$$g_{2}\left(C,P\right)=\min\left\{1-\frac{C}{H_{3}\left(C\right)},1-\frac{P}{H_{4}\left(P\right)}\right\}.$$

The coefficient functions $H_{i}$ are the piecewise linear functions

$$\begin{array}{ccc} H_{1}\left(C\right) & = & k_{7}*H\left(k_{7}-C\right)+C*H\left(C-k_{7}\right)*H\left(k_{8}-C\right)+k_{8}*H\left(C-k_{8}\right)\\ H_{2}\left(P\right) & = & k_{9}*H\left(k_{9}-P\right)+P*H\left(P-k_{9}\right)*H\left(k_{10}-P\right)+k_{10}*H\left(P-k_{10}\right)\\ H_{3}\left(C\right) & = & k_{3}*H\left(k_{3}-C\right)+C*H\left(C-k_{3}\right)*H\left(k_{4}-C\right)+k_{4}*H\left(C-k_{4}\right)\\ H_{4}\left(P\right) & = & k_{5}*H\left(k_{5}-P\right)+P*H\left(P-k_{5}\right)*H\left(k_{6}-P\right)+k_{6}*H\left(P-k_{6}\right), \end{array}$$

where

$$0<k_{3}<k_{4},\;0<k_{5}<k_{6},\;0<k_{7}<k_{8},\;0<k_{9}<k_{10}.$$

Here the function *H* is defined by

$$H\left(x\right)=\left\{\begin{array}{cc} 1, & \text{if}\;x>0,\\ \frac{1}{2} & \text{if}\;x=0,\\ 0, & \text{if}\;x<0. \end{array}\right.$$

**Figure 1:**
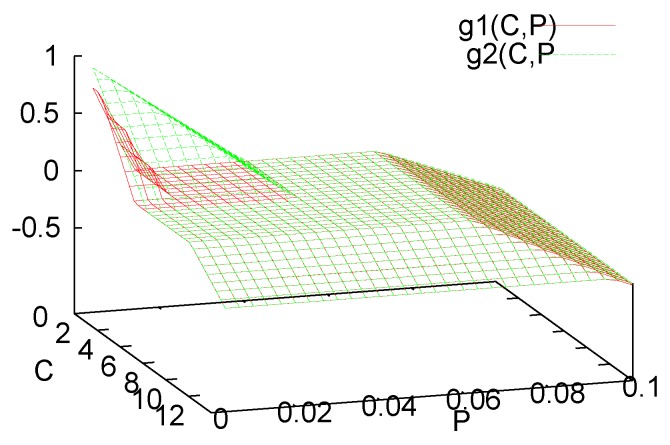
Piecewise linear, continuous net growth rate $g_{i}\left(C,P\right)$: The population grows, g>0, for small values of *C* and *P* and decays, g<0, if either *C* or *P* becomes large; the region in between marks the neutral, stationary phase.

These growth functions are sketched in Figure 1. Since *L. lactis* is more tolerant to high values of *C* and *P* than *L. monytogenes*, we may assume that $g_{1}\left(C,P\right)\leq g_{2}\left(C,P\right)$. Furthermore we may assume that the saturation levels for production of *C* and *P* are in the decay range,

(6) $$k_{1}>k_{8},\;k_{2}>k_{4},\;k_{11}>\max\left\{k_{6},k_{10}\right\}$$

The piecewise definition of the reaction kinetics is unusual in the context of Mathematical Biology in general and microbial growth modeling in particular, where normally sufficiently smooth response functions are the assumed. However, because these functional descriptions, along with quantitative parameters were identified from experiments [2,14], we chose to use them as reported in the experimental literature, rather than mollifying them for mathematical convenience, even if this comes at the expense of increased algebraic complexity.

The right hand side of our model (1)–(5) is continuous but not everywhere differentiable, due to the piecewise definition of $g_{1}\left(C,P\right)$ and $g_{2}\left(C,P\right)$. However, the one-sided derivatives of $g_{1}\left(C,P\right)$ and $g_{2}\left(C,P\right)$ exist and are bounded from below by negative numbers that depend on $k_{i}$, i=3,…,10 (note that $g_{1,2}\left(C,P\right)$ are monotonously decreasing functions). In particular we have, because of $g_{1}\left(C,P\right)\leq g_{2}\left(C,P\right)$, that

$$\max\left|\frac{\partial g_{1}\left(C,P\right)}{\partial C}\right|=\max\left\{\frac{1}{k_{7}},\frac{1}{k_{8}}\right\}=\frac{1}{k_{7}},$$

$$\max\left|\frac{\partial g_{1}\left(C,P\right)}{\partial P}\right|=\max\left\{\frac{1}{k_{10}},\frac{1}{k_{9}}\right\}=\frac{1}{k_{9}},$$

$$\max\left|\frac{\partial g_{2}\left(C,P\right)}{\partial C}\right|=\max\left\{\frac{1}{k_{3}},\frac{1}{k_{4}}\right\}=\frac{1}{k_{3}},$$

$$\max\left|\frac{\partial g_{2}\left(C,P\right)}{\partial P}\right|=\max\left\{\frac{1}{k_{5}},\frac{1}{k_{6}}\right\}=\frac{1}{k_{5}}.$$

Thus a Lipschitz constant can be found such that (1)–(5) satisfies a Lipschitz condition, which guarantees unique solutions to the initial value problem. These solutions are continuous and at least once differentiable.

**Proposition** **1.**  *The solutions to (1)–(5) with non-negative initial data are bounded from above and* $C,N_{1},N_{2},M$ *remain non-negative.*

**Proof.**  First we remark, by comparison with the trivial solution N(t)≡0 of (1) and (2), that the bacterial population sizes $N_{1}\left(t\right)$ and $N_{2}\left(t\right)$ are non-negative.

From (5) it follows $\max\{M\left(0\right),M_{0}\}\geq M\left(t\right)\geq 0$ by comparison, because $N_{2}\geq 0$.

Let $\bar{C}:=\max\left\{k_{1},k_{2},C_{0}\right\}$. Then it follows from (3), because of $N_{1}\geq 0,\;N_{2}\geq 0$, and again by comparison that $C\left(t\right)\leq C_{0}$. Similarly, let $\hat{C}:=\min\left\{k_{1},k_{2},C_{0},C\left(0\right)\right\}$ to obtain $C\left(t\right)\geq \hat{C}$; moreover, $\min\left\{k_{1},k_{2},C_{0}\right\}\leq C\left(t\right)\leq \max\left\{k_{1},k_{2},C_{0}\right\}$ for all sufficiently large *t*.

From (4) it follows by comparison with $\bar{P}:=\max\left\{k_{11},P_{0}\right\}$ that $\bar{P}\geq P\left(t\right)$.

Thus we have established that $g_{1}\left(C,P\right)\geq g_{1}\left(\bar{C},\bar{P}\right)$ and $g_{2}\left(C,P\right)\geq g_{2}\left(\bar{C},\bar{P}\right)$. This allows us to improve our lower estimates on the population sizes $N_{1}\left(t\right)\geq N_{1}\left(0\right)e^{(\mu_{1}g_{1}\left(\bar{C},\bar{P}\right)-q)t}$ and $N_{2}\left(t\right)\geq N_{2}\left(0\right)e^{(\mu_{1}g_{1}\left(\bar{C},\bar{P}\right)-q)t}$. More important, however, is that due to (6) we have $g_{1}\left(\bar{C},\bar{P}\right)<0$ and $g_{2}\left(\bar{C},\bar{P}\right)<0$, which implies that $N_{1}$ and $N_{2}$ are bounded form above. To see this, we assume that this statement does not hold for one of them, say we assume that $N_{1}\to \infty$ (the same line of argumentation applies to $N_{2}$). Then due to (3), $C\to k_{1}$. Due to (6), this would imply that $N_{1}$ is eventually declining, which is a contradiction to our assumption. Because of continuity this means that $N_{1}$ has a maximum, i.e., is bounded. ☐

**Remark** **1.**  *The non-negativity of P cannot be established with standard arguments without additional restrictions on parameters. Indeed, one can construct non-negative initial data such that P becomes negative. This situation obviously marks a breakdown of the model. Recall, however, that* P→0 *means* pH→∞, *wherefore for practical purposes this break down is not relevant. In none of the numerous simulations that we ran for our study did this breakdown situation occur or did solutions come close to this breakdown situation.*

The mathematical theory of competition in the chemostat and related laboratory devices, such as gradostats, is well understood [21]. However, the underlying mathematical machinery makes heavy use of certain close relationships between bacterial growth rates and rates of substrate consumptions, which do not hold for the biocontrol model (1)–(5). Therefore, the mathematical results from traditional chemostat analysis cannot be carried over in a straightforward manner.

The model (1)–(5) is a five-dimensional nonlinear autonomous system of differential equations. For all practical purposes models of this type are typically difficult to study with the qualitative methods of dynamics systems theory if they do not have certain useful properties, such as quasi-monotonicity etc. Moreover, our model has 21 parameters, which could be somewhat reduced by 4 after non-dimensionalization. Furthermore, because of the piecewise definition of $g_{1}\left(C,P\right)$, $g_{2}\left(C,P\right)$, the Jacobian is only piecewise defined and only for $C\neq k_{3,4,7,8}$ and $P\neq k_{5,6,9,10}$. A rigorous mathematical analysis would require us to distinguish between a multitude of cases, depending on parameters. The resulting algebraic expressions will be too complex to lend themselves to biologically insightful results. Instead, we will embark on studying the model behavior with a mix of analytical arguments and computational techniques.

In [2] a complete set of reaction parameters is given, that was derived from laboratory experiments and model simulations of the competitive growth of *L. monocytogenes* and *L. lactis* in vegetable broth. We will use these data in all our simulations. They are summarized in Table 1. Note that the less tolerant pathogen grows faster than the control agent, $\mu_{2}>\mu_{1}$. This leaves us with four unknown parameters that were introduced by the chemostat description, flow rate *q* and the bulk concentrations $C_{0},P_{0},M_{0}$. In our studies we pick the latter to be $M_{0}=4mM$, and explore the system behavior with respect to the remaining parameters $q,C_{0},P_{0}$.

We assume for our simulations that the reactor is initially filled with the same liquid that is used as bulk liquid. This fixes the initial data

(7) $$C\left(0\right)=C_{0},\;P\left(0\right)=P_{0},\;M\left(0\right)=M_{0}.$$

## 3. Analysis of the Single Species Pathogen Sub-Model

Before we study the complete dual-species model, in order to gain a better insight into self-limiting growth behavior, we study a simpler single species model, namely system (1)–(5) for $N_{2}\left(0\right)=0$. This is the model for the uncontrolled pathogen. It is easy to verify that then $N_{2}\left(t\right)\equiv 0$ for all t>0 and the model reduces to

(8) $$\begin{array}{ccc} \frac{dN_{1}}{dt} & = & \mu_{1}g_{1}\left(C,P\right)N_{1}-qN_{1}, \end{array}$$

(9) $$\begin{array}{ccc} \frac{dC}{dt} & = & \gamma N_{1}\left(1-\frac{C}{k_{1}}\right)+q\left(C_{0}-C\right), \end{array}$$

(10) $$\begin{array}{ccc} \frac{dP}{dt} & = & \rho C\left(1-\frac{P}{k_{11}}\right)+q\left(P_{0}-P\right). \end{array}$$

In order to investigate whether species $N_{1}$ persists or dies out, we investigate the stability of the equilibrium

(11) $${N_{1}}^{*}=0,\;C^{*}=C_{0},\;P^{*}=\frac{\rho C_{0}+qP_{0}}{\frac{\rho C_{0}}{k_{11}}+q}.$$

The Jacobian in this point is obtained as the triangular matrix

$$J^{*}=\left(\begin{array}{ccc} \mu_{1}g_{1}\left(C_{0},P^{*}\right)-q & 0 & 0\\ \gamma \left(1-\frac{C^{*}}{k_{1}}\right) & -q & 0\\ 0 & \frac{q(k_{11}-P_{0})}{\rho C_{0}+qk_{11}} & -\left(\frac{\rho C_{0}}{k_{11}}+q\right) \end{array}\right).$$

The last two eigenvalues $\lambda_{2}=-q$ and $\lambda_{3}=-\left(\frac{\rho C_{0}}{k_{11}}+q\right)$ are always negative. The sign of the first eigenvalue $\lambda_{1}=\mu_{1}g_{1}\left(C^{*},P^{*}\right)-q$ depends on the model parameters. We note that for large enough flow rates *q* the steady state value $P^{*}$ converges to the bulk concentration $P_{0}$, while for small *q* it converges to the saturation concentration $k_{11}$. For realistic parameters we have $g(C_{0},k_{11})<0$, i.e., we expect for small enough *q* that $\lambda_{1}<0$, i.e., the population dies out due to self inhibition at high *P* values. On the other hand, the concentrations $C_{0}$ and $P_{0}$ in the growth medium are usually small enough that $g(C_{0},P_{0})>0$. Thus for high enough flow rates the population dies out due to washout.

This can be formulated more precisely by substituting $P^{*}$ into $\lambda_{1}$, which leads to the following quadratic inequality for the persistence of species $N_{1}$

$$k_{11}{\left(\frac{q}{\mu }\right)}^{2}+\frac{q}{\mu }\left(\frac{\rho C_{0}}{\mu }-k_{11}+\frac{k_{11}}{k_{9}}P_{0}\right)+\frac{\rho C_{0}}{\mu }\left(\frac{k_{11}}{k_{9}}-1\right)<0.$$

From this we find that $\lambda_{1}$ is positive if

(12) $$q_{1}:=\frac{\rho C_{0}}{\mu k_{11}}\left(\frac{k_{11}}{k_{9}}-1\right)<q<\mu g_{1}\left(C_{0},P_{0}\right)=:q_{2},$$

and negative if one of the inequalities is reversed.

**Figure 2:**
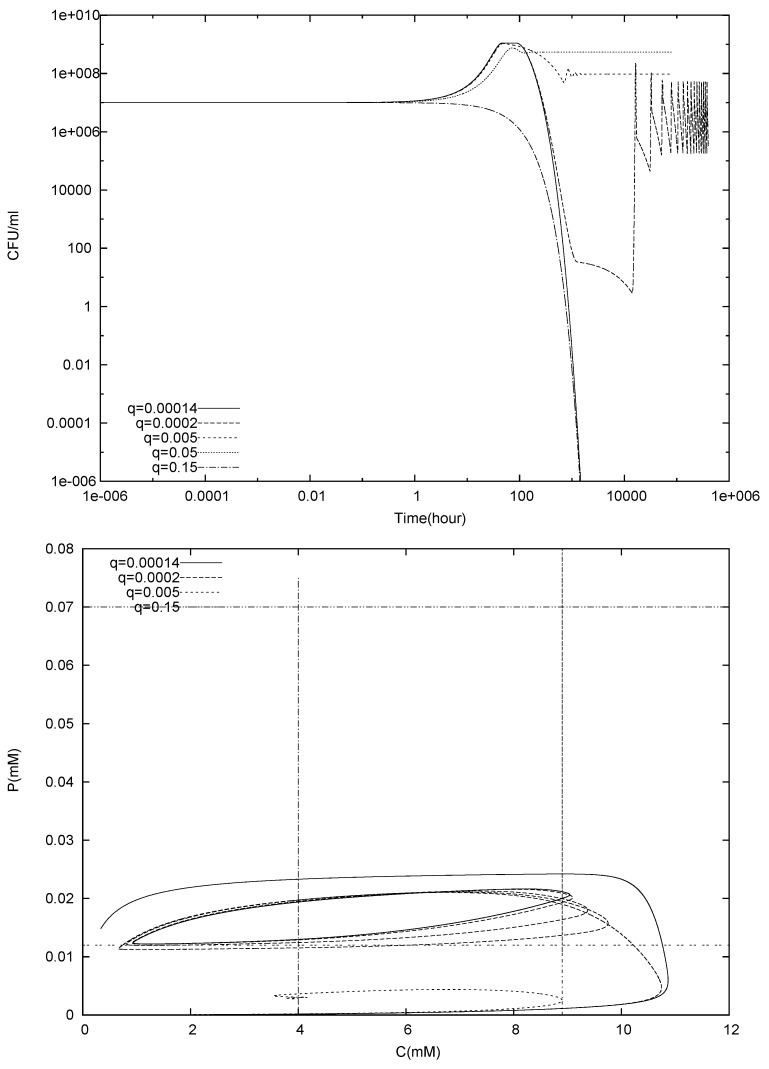
Simulation of the single species model (8)–(10) for various flow rates *q*. **Top panel**: population size $N_{1}\left(t\right)$; **Bottom panel:** lactic acid concentration C(t) and hydrogen ion concentration P(t) in the *C*-*P*-plane. The vertical and horizontal lines at $C=k_{7}$ and $C=k_{8}$ and $P=k_{9}$ and $P=k_{10}$ mark the transition from growth to neutral to decay regimes.

In Figure 2 we show simulations of (8)–(10), using the parameters from Table 1. The bulk concentration $P_{0}=10^{-5}$ and $C_{0}=0.1$ were chosen small enough to permit growth of the bacteria under ideal conditions. The initial conditions for *C* and *P* were chosen as the bulk concentration values, for $N_{1}\left(0\right)$ we chose 10^7^ CFU/mL. The simulations were conducted for various flow rates *q*, ranging from q=0.00014 to q=0.15. For the highest of these values $q>q_{2}$, whence (12) predicts washout, for the smallest value $q<q_{1}$, whence (12) predicts extinction due to self-inhibition.

This is confirmed in the top panel of Figure 2. For the largest flow rate, the flow dominates growth and the population size $N_{1}\left(t\right)$ decreases monotonically. On the other hand, for the smallest flow rate, the population initially grows, reaches a plateau and then eventually dies out. This is explained by the bottom panel of Figure 2. For the highest flow rate, C, P remain in the growth region throughout, but growth is dominated by washout. For the smallest flow rate, however, we notice that initially both *C* and *P* increase as a consequence of bacterial activity. They first reach and pass through the neutral range and then, as *C* continues to be produced, reach the decay range. Eventually, when $N_{1}$ is small enough, the second term in (9) dominates over the first one and *C* declines. After some time also *P* starts to decline and the system converges to the equilibrium (11) with $g_{1}\left(C_{0},P^{*}\right)=0$.

The behaviour for intermittent values of the flow rate $q_{1}<q<q_{2}$ can vary. In all tested cases, the population establishes itself, often after passing through a brief oscillatory phase which occurs when C, P reach the decay range first and then return to growth range as a consequence of a declining bacterial population. The longterm behavior in these simulations is independent of the initial concentration for $N_{1}$, although the transients might change.

The case q=0.0002 requires some additional explanation. The criterion (12) predicts that the population will eventually establish itself. This is also confirmed in Figure 2. The population increases first, reaches a plateau, decreases, then increases again and starts oscillating, due to *C* and *P* oscillating between the growth and decay stage. For all practical purposes, however, we point out that at around t=1000 the population drops down to 10 CFU/mL, i.e., to a level where the description of the bacterial population by a continuous variable breaks down. Thus, while the numerical simulation confirms the theoretical analysis, the model is not valid anymore, and does not allow a conclusion whether the theoretical result is rigorous from a practical point of view.

## 4. Numerical Experiments for the Complete Dual Species Biocontrol Model

### 4.1. Batch Cultures

We start with a simple simulation experiment of batch cultures, i.e., the special case q=0 of our model (1)–(5). This is the case that was originally studied in [2]. We will investigate in a simple simulation experiment how adding the control agent *L. lactis* to the batch culture initially will affect the eradication of the pathogen *L. monocytogenes*.

**Figure 3:**
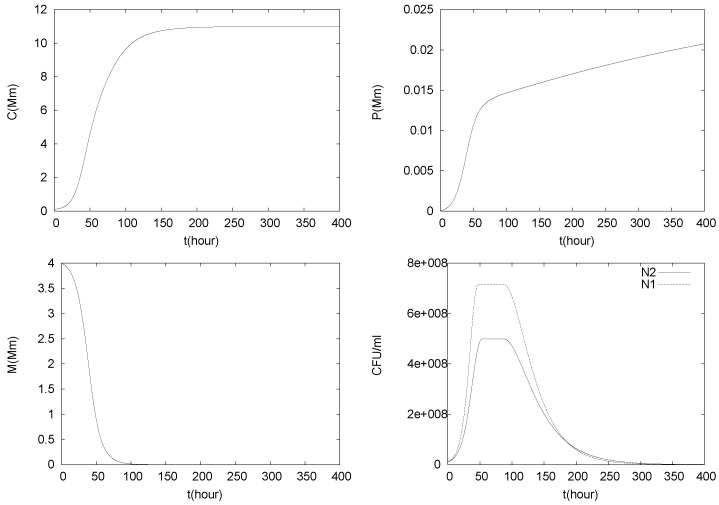
Simulation of (1)–(5) with q=0 and $N_{1}\left(0\right)=N_{2}\left(0\right)=10^{7}$ CFU/mL, $C\left(0\right)=C_{0}=0.1$ mM, $P\left(0\right)=P_{0}=0.0001$ mM and M(0)=4 mM.

A typical simulation is shown in Figure 3 for initial values

$$N_{1}\left(0\right)=N_{2}\left(0\right)=10^{7}\;\text{CFU}/\text{mL},$$

$$C\left(0\right)=C_{0}=0.1\;\text{mM},P\left(0\right)=P_{0}=0.0001\;\text{mM},M\left(0\right)=4\;\text{mM}.$$

The bacterial growth curves show the typical three stages, a growth phase initially, followed by a stationary phase, and then the decay phase [5]. Since in these simulations q=0, malate is not replenished. Therefore, *M* is continuously decreasing and eventually vanishes. On the other hand, the concentrations of lactic acids *C* and of hydrogen ions *P* increase continuously due to production, and in the absence of washout. Production of lactic acids slows down as the populations vanish and *C* reaches a plateau at saturation concentration level. Similarly, pH (i.e., *P*) reaches a stationary value eventually.

In a simple numerical experiment we investigate how the initial bacterial count of the control agent *L. lactis* affects the decay of the pathogen *L. monocytogenes*. To this end, we keep the initial data for $N_{1},\;C,\;P,\;M$ the same as in the previous simulations,

$$N_{1}\left(0\right)=10^{7}\;\text{CFU}/\text{mL},\;C\left(0\right)=C_{0}=0.1\;\text{mM},$$

$$P\left(0\right)=P_{0}=0.0001\;\text{mM},\;M\left(0\right)=4\;\text{mM},$$

but vary $N_{2}\left(0\right)$ to be

$$N_{2}\left(0\right)=kN_{1}\left(0\right)=k\cdot 10^{7}$$

for different values k=0,1,2,4,8,16,32. We measure in our simulations the decay time $t_{d}$ for $N_{1}$, which we define as the first time at which the population size $N_{1}$ falls below one percent of its initial value, i.e.,

$$t_{d}=\min\left\{t>0:N_{1}\left(t\right)\leq 0.01N_{1}\left(0\right)\right\}.$$

**Figure 4:**
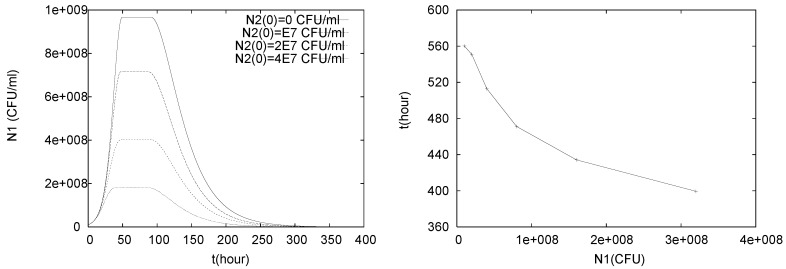
Simulation of model (1)–(5) with q=0, for initial data $N_{1}\left(0\right)=10^{7}$ CFU/mL, $N_{2}\left(0\right)=k*10^{7}$ CFU/mL, C(0)=0.1 mM, P(0)=0.0001 mM, M(0)=4 mM. The initial amount of control agent is varied by picking different values for *k*. The left plot shows the population size of *L. monocytogenes* for different initial population sizes of *L. lactis* (k=0,1,2,4). In the right figure the decay time $t_{d}$ for *L. monocytogenes* is plotted for different initial population sizes of *L. lactis* (k=1,2,4,8,16,32).

In Figure 4 we plot the population size $N_{1}\left(t\right)$ of the pathogen for different amounts of control agent, as well as $t_{d}$ as a function of the initial population size of the biocontrol agent. While we see the growth, stationary and decay phase in all simulations, it is of particular interest that the duration of the stationary phase seems to be not simply correlated with the initial amount of control agent. For example, for both k=0 and k=2 we observe slightly longer stationary phases than for k=1. The onset of the stationary phase, however, happens slightly earlier for higher initial populations of control agents, and the population level of the stationary phase clearly decreases as the initial number of control agents increases. Thus in order to keep the pathogen cell count below a certain value above initial conditions, a sufficiently high number of control agents can be added. The latter two results confirm what one might expect intuitively.

Similarly, increasing the number of control agents initially accelerates eradication of the pathogen, as expected. However, the stopping time does not decrease proportionally with the increase in the initial count of control agents. In fact doubling the number of control agents leads to a decrease in stopping time of less than 10%. Different choices of initial data for $N_{1}$ lead to qualitatively similar results.

### 4.2. Longterm Behavior of the Full Chemostat Model

As previously discussed, with the parameters in Table 1 and the assumptions on initial conditions (7), along with

$$N_{1}\left(0\right)=N_{2}\left(0\right)=10^{7}\;\text{CFU}/\text{mL}$$

and $M_{0}=4$ mM, we are left with three free parameters that can determine the behavior of the solutions of model (1)–(5), namely $C_{0},P_{0},q$. Since our focus will be on survival and persistence of bacterial populations in the chemostat, we can restrict the ranges of $C_{0}$ and $P_{0}$ to the range where both species can grow, in order to avoid a trivial outcome. Thus

(13) $$0\leq C_{0}\leq k_{3},\;0\leq P\leq k_{5}.$$

Characteristic for the chemostat setup is that the bacterial populations wash out if the flow rate *q* exceeds the growth rates, independent of the initial bacterial concentration. This is expressed by the asymptotic stability of the trivial steady state, which for our system is obtained as

$$E_{0}=\left(0,0,C^{*},P^{*},M^{*}\right)$$

with

$$C^{*}=C_{0},\;P^{*}=\frac{\rho C_{0}+qP_{0}}{\frac{\rho C_{0}}{k_{11}}+q},\;M^{*}=M_{0},$$

where again for small enough *q* we find $P^{*}\approx k_{11}$ and for large *q* we have $P^{*}\approx P_{0}$. For complete washout to occur, we require in extension of the single species model above that

$$q>\mu_{1}g_{1}\left(C^{*},P^{*}\right)\;\text{and}\;q>\mu_{2}g_{2}\left(C^{*},P^{*}\right).$$

Indeed, the sign pattern of the Jacobian in this equilibrium point is then

$$J\left(E_{0}\right)=\left(\begin{array}{ccccc} - & 0 & 0 & 0 & 0\\ 0 & - & 0 & 0 & 0\\ + & + & - & 0 & 0\\ 0 & - & + & - & 0\\ 0 & - & 0 & 0 & - \end{array}\right),$$

which implies asymptotic stability of $E_{0}$. Note that this is in particular true for $q>q_{\infty }:=\max\left\{\mu_{1},\mu_{2}\right\}$, due to the monotonicity of $g_{1}\left(C,P\right)$ and $g_{2}\left(C,P\right)$. Therefore, we can restrict ourselves to

(14) $$0\leq q\leq q_{\infty }.$$

**Figure 5:**
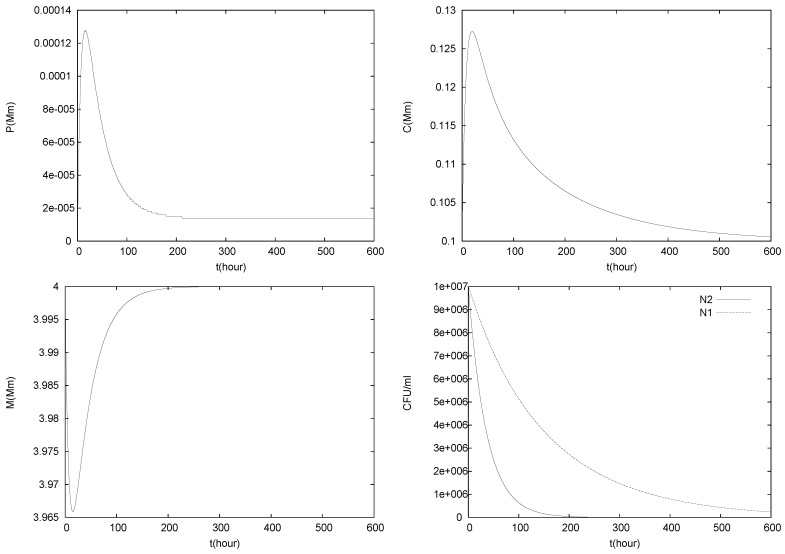
Simulation of model (1)–(5), with $C_{0}=0.1mM,\;P_{0}=0.00001mM$ and q=0.13. Note that $q<q_{\infty }$ but $q>\mu_{1,2}g_{1,2}\left(C^{*},P^{*}\right)$.

**Figure 6:**
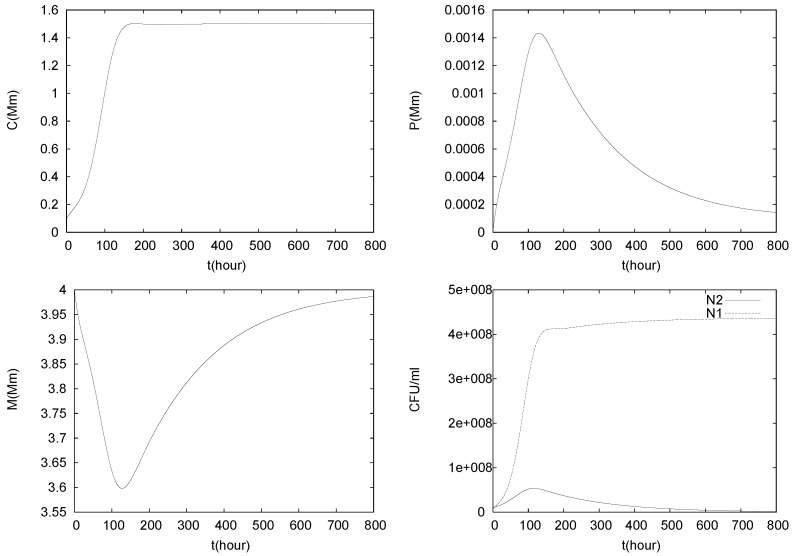
Simulation of model (1)–(5), with $C_{0}=0.1mM,\;P_{0}=0.00001mM$ and q=0.08.

**Figure 7:**
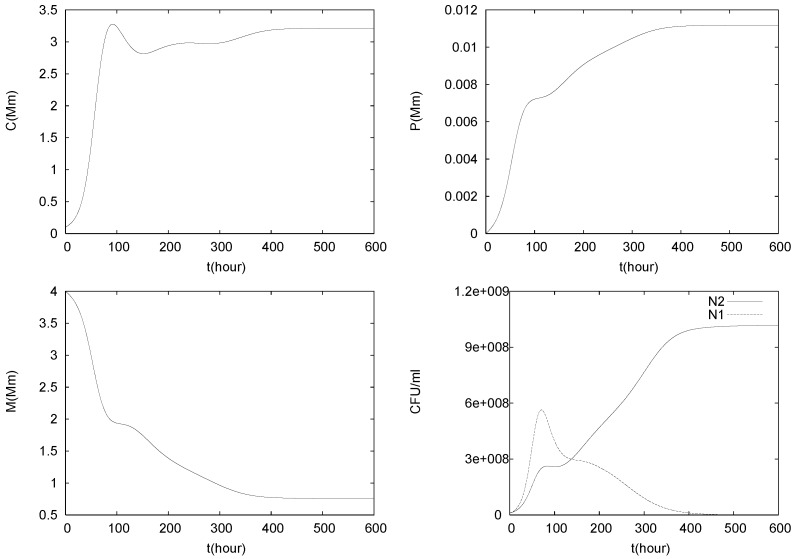
Simulation of model (1)–(5), with $C_{0}=0.1mM,\;P_{0}=0.00001mM$ and $q=0.04<q^{*}$.

The results of three simulations of (1)–(5) with $C_{0}=0.1$ mM, $P_{0}=0.00001$ mM and different values for the flow rate *q* are shown in Figure 5, Figure 6 and Figure 7.

In Figure 5, the flow rate *q* is smaller than $q_{\infty }$ but bigger than the growth rates at equilibrium concentrations, $\mu_{1}g_{1}\left(C^{*},P^{*}\right)$ and $\mu_{2}g_{2}\left(C^{*},P^{*}\right)$. Thus, *q* is big enough to induce washout of both species. $N_{1}\left(t\right)$ and $N_{2}\left(t\right)$ are strictly decreasing functions and equilibrium $E_{0}$ is attained. The malate concentration decreases first, while sufficient $N_{2}$ is in the system for degradation but then goes back to $M_{0}$ due to replenishment. Both *P* and *C* first increase due to production by the bacteria and then drop to their equilibrium values when the bacterial populations vanish.

In Figure 6, the flow rate is decreased to q=0.08, i.e., it is smaller than both maximum growth rates. Initially growth conditions are favorable for both species, which leads to growth of $N_{1}$ and $N_{2}$, implying an increase of *C* and *P*, as well as a drop in *M*. While *L. lactis* is more tolerant than *L. monocytogenes*, it also has a smaller maximum growth rate, $\mu_{2}<\mu_{1}$. Thus, eventually, the actual growth rate $\mu_{2}g\left(C,P\right)$ of the control agent, while still positive, drops below the dilution rate *q* due to growth in *C* and *P* and $N_{2}$ dies out. After the extinction of $N_{2}$, the malate concentration goes back to its bulk value. The system eventually attains a steady state of type

$$E_{1}=\left(N_{1}^{*},0,C^{*},P^{*},M_{0}\right),$$

with

$$C^{*}=\frac{qC_{0}+\delta N_{1}^{*}}{\frac{\delta N_{1}^{*}}{k_{2}}+q},\;P^{*}=\frac{\rho C^{*}+qP_{0}}{\frac{\rho C^{*}}{k_{11}}+q}.$$

Thus, the less tolerant but faster growing pathogen survives. In Figure 7 the flow rate is further decreased to q=0.04. Again, initially both populations begin to grow. Compared to the previous case in Figure 6, the population sizes reach higher level and accordingly also *C* and *P* grow faster. Hence, the growth conditions become more unfavorable and lead to the extinction of the less tolerant pathogen *L. monocytogenes*, while the more tolerant control agent *L. lactis* has the opportunity to establish itself, despite its lower growth rate but due to the also lower flow rate. With $N_{2}$ taking a plateau, the malate concentration declines. Eventually the system reaches an equilibrium of the form

$$E_{2}=\left(0,N_{2}^{*},C^{*},P^{*},M^{*}\right),$$

with

$$C^{*}=\frac{qC_{0}+\gamma N_{2}^{*}}{\frac{\gamma N_{2}^{*}}{k_{1}}+q},\;P^{*}=\frac{-\kappa \theta N_{2}^{*}M^{*}+\rho C^{*}+qP_{0}}{\frac{\rho C^{*}}{k_{11}}+q},\;M^{*}=\frac{qM_{0}}{\theta N_{2}^{*}+q}.$$

Thus, the control agent inhibits the establishment of the pathogen.

A fourth type of steady state that is admitted by model (1)–(5) is the co-existence equilibrium in which $N_{1}^{*}>0$, and $N_{2}^{*}>0$,

$$E_{3}=\left(N_{1}^{*},N_{2}^{*},C^{*},P^{*},M^{*}\right).$$

In this case

(15) $$\mu_{1}g_{1}\left(C^{*},P^{*}\right)=\mu_{2}g_{2}\left(C^{*},P^{*}\right)=q.$$

Note that this condition of two equations for two unknowns $C^{*},\;P^{*}$ and given *q* does not have a unique solution. Since *L. lactis* is more tolerant than *L. monocytogenes*, it does not exist if $\mu_{1}>\mu_{2}$. i.e., it exists at most if the less tolerant pathogen has a higher maximum growth rate than the more tolerant control agent, $\mu_{2}>\mu_{1}>q$. If $C^{*},\;P^{*}$ exist that satisfy (15), then there are infinitely many such pairs, thus the calculation of $C^{*},\;P^{*}$ can not be decoupled from the rest of the system when calculating the equilibrium points. One obtains

$$C^{*}=\frac{qC_{0}+\delta N_{1}^{*}+\gamma N_{2}^{*}}{\frac{\delta N_{1}^{★}+\gamma N_{2}^{★}}{k_{1}}+q},\;P^{*}=\frac{-\kappa \theta N_{2}^{★}M^{★}+\rho C^{★}+qP_{0}}{\frac{\rho C^{★}}{k_{11}}+q},\;M^{*}=\frac{qM_{0}}{\theta N_{2}^{★}+q},$$

which still depend on the yet undetermined $N_{1}^{*},\;N_{2}^{*}$. In principle, these can be determined from plugging $C^{*}$ and $P^{*}$ into the piecewise defined equations (15), however, the resulting expression are too unwieldy to be of practical value. The same holds for the Jacobian and its eigenvalues. Its sign pattern is

$$J\left(E_{3}\right)=\left(\begin{array}{ccccc} 0 & 0 & - & - & 0\\ 0 & 0 & - & - & 0\\ + & + & - & 0 & 0\\ 0 & - & + & - & -\\ 0 & - & 0 & 0 & - \end{array}\right).$$

To the best of our knowledge no sign pattern criterion similar to those proposed in [27,28,29] exists that could be applied to this matrix to reach conclusions about stability or instability. Numerical calculations for some choices of parameters $C_{0},P_{0},q$ indicate a positive eigenvalue, and thus, instability.

The results in Figure 5, Figure 6 and Figure 7 show that the flow rate *q* can play a crucial role in (1)–(5), which is a consequence of a delicate balance between (i) replenishment of the reactor with fresh, growth permitting medium; (ii) bacterial population dynamics, and (iii) removal of bacteria from the reactor. Of these three, the first and last clearly are primarily controlled by *q* in the sense that both effects become stronger as *q* increases. These results on longterm behaviour are independent of initial bacterial counts, which however could affect transients, i.e., how quickly the equilibrium solutions are approached.

**Figure 8:**
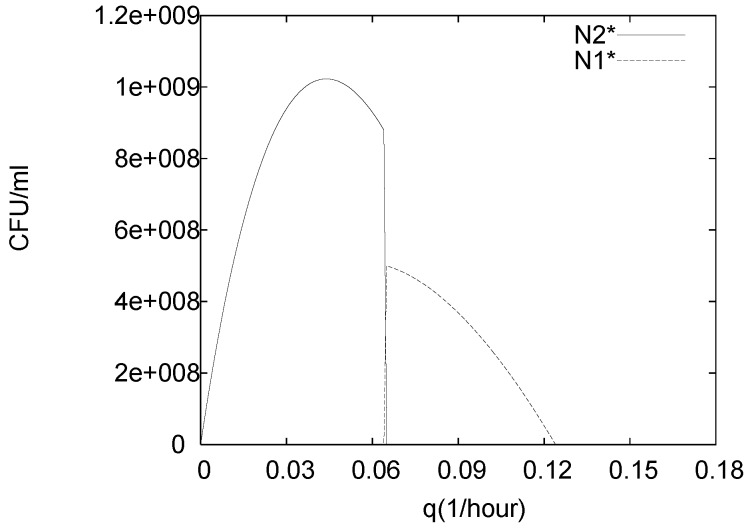
Population sizes $N_{1}^{*}$ and $N_{2}^{*}$ at steady state for model (1)–(5) for varying flow rate *q* and $C_{0}=0.1mM,\;P_{0}=0.00001mM$.

In order to illustrate the dependency of the long term behaviour on the flow rate, we solve (1)–(5) for *q* varying over the interval $0\leq q\leq q_{\text{max}}$, where $q_{\text{max}}$ is chosen large enough to include washout steady states, $q_{\text{max}}>q_{\infty }=\max\left\{\mu_{1},\mu_{2}\right\}$. In Figure 8 the population sizes $N_{1}^{*}$ and $N_{2}^{*}$ at steady state are plotted in dependence of *q*. The case q=0 is the case that was studied in the previous Section 4.1. As discussed there, the populations grow and induce unfavorably high concentrations of *C* and *P* which first lead to self-inhibition and eventually to decay. This is primarily a consequence of the growth medium not being replenished but also of the fact that active cells are not removed, which increases *C* and thus *P*. When increasing *q* to a critical value $q^{*}\approx 0.065$, the control agent *L. lactis* survives while the pathogen *L. monogytogenes* dies out, i.e., an equilibrium of type $E_{2}$ is reached. Initially the steady state population size of the control agent $N_{2}^{*}$ increases until q≈0.04. This is primarily a consequence of increasing the flow rate at which fresh medium is replenished, i.e., controlled by (i). With the increasing population size also *C* and *P* increase; only if *q* becomes large enough to contribute considerably to the washout of the *L. lactis* the steady state population size decreases again. Thus, for q>0.04 the longterm behavior is dominated by (iii). As *q* passes through $q^{*}$, the more tolerant but slower growing control agent is not viable anymore; the growth rate $\mu_{2}g_{2}\left(C^{*},P^{*}\right)$ falls below *q* and the control agent is washed out. This allows the less tolerant but faster growing pathogen $N_{1}$ to establish itself as long as $\mu_{1},g_{1}\left(C^{*},P^{*}\right)>q$, i.e., an equilibrium of type $E_{1}$ is attained. Note that also $C^{*}$ and $P^{*}$ change with *q*. Hence, (iii) is stronger than (i). However, due to the dominance of (iii), increasing *q* further leads to decreasing steady state populations $N_{1}^{*}$, until complete washout occurs and both populations die out and an equilibrium of type $E_{0}$ is reached. Equilibria of type $E_{3}$ are never attained in our simulations. For the transition case from $E_{1}$ to $E_{2}$ the eigenvalues of $E_{3}$ were computed and it was found that one of them is positive, i.e., $E_{3}$ is unstable.

**Figure 9:**
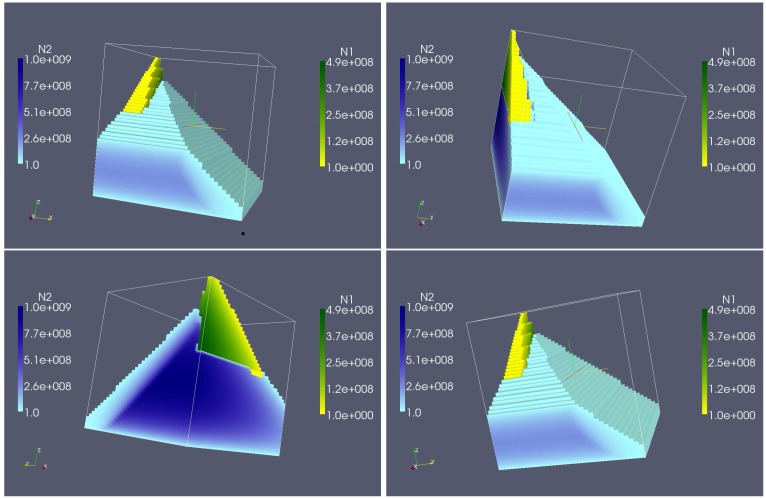
Exploration of the three dimensional parameter space *q* (*x*-axis), $C_{0}$ (*y*-axis), $P_{0}$ (*z*-axis). Shown are the sizes of the microbial populations $N_{1}$ and $N_{2}$ at steady state (4 different views of the same simulation data set).

So far all computations were carried out for more or less arbitrarily fixed small model parameters $C_{0}$ and $P_{0}$. These bulk concentrations of the growth inhibitors were chosen small enough so that it can be anticipated that bacterial communities can establish themselves. The calculations above show that the steady state values explicitly depend on $C_{0}$ and $P_{0}$. Therefore, in order to investigate whether the behavior seen in Figure 8 is generally observable, we repeat the simulations for various bulk concentration values. To this end the region given by $0\leq C_{0}\leq k_{3}$ and $0\leq P_{0}\leq k_{5}$ is discretized by a regular grid of dimension 39 × 37 and for each point $\left(C_{0},P_{0}\right)$ on this grid the model was solved for 48 different values of *q* between $0<q\leq q_{max}$. Thus in total, model (1)–(5) was solved 69,264 times for different parameter combinations of $C_{0},P_{0},q$. In Figure 9 the steady state population sizes $N_{1}^{*}$ and $N_{2}^{*}$ that are reached in these simulations are visualized. The values $N_{1}^{*}$ for the pathogen *L. monocytogenes* are color coded using a yellow-green map, while the values $N_{2}^{*}$ for the control agent *L. lactis* are color coded in a blue-scale color map. The behavior that was seen in Figure 8 is only obtained for relatively small values of $C_{0}$ and $P_{0}$: for small values of *q* the control agent survives while the pathogen cannot establish itself. After *q* passes through a critical value $q^{*}$, which depends on $C_{0}$ and $P_{0}$, the control agent is washed out and the pathogen can establish itself until *q* is finally large enough to wash it out, too. Note that the washout value for *q* also depends on $C_{0}$, and $P_{0}$. If $C_{0}$ and $P_{0}$ are chosen larger, the environmental conditions are too unwieldy for the pathogen to survive. For small flow rates the more tolerant control agent can establish itself but it is washed out if *q* becomes large. The less favorable the bulk conditions are, the smaller is the minimum flow rate that induces washout.

### 4.3. Continuously Adding Control Agents to Eradicate the Pathogens

The question arises naturally whether the addition of control agents to the system will enable a quicker eradication of the pathogen. To study this question, the model (1)–(5) is slightly modified by adding a supply term to (2). We obtain

(16) $$\begin{array}{ccc} \frac{dN_{1}}{dt} & = & \mu_{1}g_{1}\left(C,P\right)N_{1}-qN_{1} \end{array}$$

(17) $$\begin{array}{ccc} \frac{dN_{2}}{dt} & = & \mu_{2}g_{2}\left(C,P\right)N_{2}+q\left(N_{2}^{0}-N_{2}\right) \end{array}$$

(18) $$\begin{array}{ccc} \frac{dC}{dt} & = & \gamma N_{1}\left(1-\frac{C}{k_{1}}\right)+\delta N_{2}\left(1-\frac{C}{k_{2}}\right)+q\left(C_{0}-C\right) \end{array}$$

(19) $$\begin{array}{ccc} \frac{dP}{dt} & = & \rho C\left(1-\frac{P}{k_{11}}\right)-\kappa \theta N_{2}M+q\left(P_{0}-P\right) \end{array}$$

(20) $$\begin{array}{ccc} \frac{dM}{dt} & = & -\theta N_{2}M+q\left(M_{0}-M\right), \end{array}$$

where the new parameter $N_{2}^{0}$ is the amount of control agents added continuously to the system. The rigorous qualitative analysis of this model is as cumbersome and impractical as it was for the original model (1)–(5). Therefore, we will restrict ourselves a priori to a computational study and report here the results. The interesting question is how the dosage amount $N_{2}^{0}$ affects eradication times of $N_{1}$.

**Figure 10:**
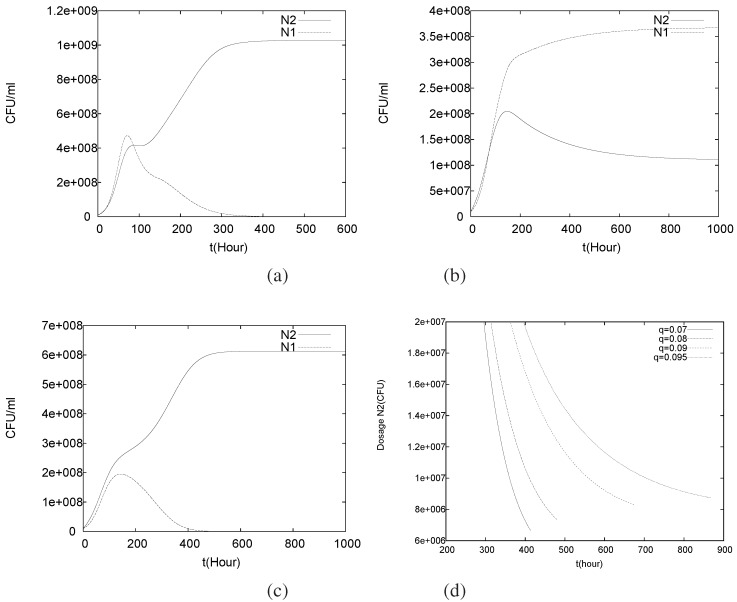
Population dynamics for the control model (16)–(20) with continuously added control agents. For (**a**) small values of $q<q^{*}$; and values $q>q^{*}$ for (**b**) small and (**c**) larger amount of control agents added; In (**d**) eradication time is plotted as a function of the dosage value of the control agent $N_{2}^{0}$ for various $q>q^{*}$.

Again we carry out the simulations for the case $C_{0}=0.1$ mM, $P_{0}=0.00001$ mM as above, but vary *q*. First we note that the longterm behavior can depend on both *q* and $N_{2}^{0}$. For small $q<q^{*}$, the pathogen could not establish itself even in the absence of control agents being added, and this is of course what one expects and finds in the new simulations, cf. Figure 10a. For flow rates $q>q^{*}$ the long term behavior depends on $N_{2}^{0}$ and $q^{*}$. For small values of $N_{2}^{0}$ coexistence of pathogen and control agent can be observed, see Figure 10b. This corresponds to the previous case where the pathogen only survived. If the value of $N_{2}^{0}$ is sufficiently increased, the pathogen dies completely out, cf. Figure 10c, i.e., it can be controlled.

In Figure 10d finally we plot the eradication time $t_{d}$ as a function of $N_{2}^{0}$ for four different flow rates $q>q^{*}$. We notice that continuously adding a constant amount of control agents becomes more effective the higher the flow rate *q*, in the sense that for the lowest flow rate the eradication time reduces by approximately 20% while for the highest flow velocity it decreases by approximately 47% if the dosage is increased from the initial population $N_{2}\left(0\right)$ to twice its value $2N_{2}\left(0\right)$. Moreover, the simulations indicate that increasing the dosage more, to values greater than $2N_{2}\left(0\right)$ will not lead to considerably shorter eradication times. Eradication times will quantitatively be different for different initial values of $N_{1}$ but qualitatively these findings carry over. In applications one will try to balance the dosage between as high as necessary to see the desired effect and to keep it as low as possible, for reasons of process performance, economical cost and to avoid negative microbial side effects that might be introduced by the control agent. The latter could be aspects of microbial food safety in the context of food preservation or texture in the context of functional foods where such a control mechanism could be used, e.g., as probiotics in dairy products.

## 5. Conclusions

Compared to mathematical models of batch cultures, the chemostat setting has the advantage that it allows a focus on the method of interaction between species, minimizing the effect of the initial population sizes. The well established mathematical theory of the chemostat [21] applies in the first place to systems of microbial competition for common but limited resources. While its qualitative results are powerful and can be considered one of the greatest success stories in Mathematical Biology, they are based on certain functional relationships between bacterial growth terms and substrate consumption terms. However, mathematical models of microbial co-culture systems that are based on other ecological interactions than competition for foods may not have these properties. Therefore, the general theory of the chemostat usually cannot be readily applied and each such system must be studied individually, cf. [20,22,23,24,30]. This is also the case for the amensalistic biopreservation model for the control of the foodborne pathogen *L. monocytogenes* by the lactic acid bacteria *L. lactis* that is studied in this paper for nutrient rich environments where competition between both species for nutrients is not growth limiting. Due to the algebraic complexity of the five-dimensional system of ordinary differential equations with 21 parameters, it was necessary to conduct the model study computationally.

Our simulations imply that the persistence of the pathogen depends on the characteristics of the growth medium and on the dilution rate. In order for the pathogen to persist, the concentrations of the detrimental substances must be low enough to allow for bacterial growth and the flow rate must be small enough to not washout the cells before they can reproduce. Only in an intermediate range of the flow rate the pathogen can survive. This is due to the more tolerant but slower growing control agent being washed out of the system in this regime. At lower flow rates the pathogen is crowded out by the control agent which is generally regarded as safe for humans. This amensalistic control effectively enhances and accelerates the self-inhibition that we observe in the uncontrolled system. At higher flow velocities, both species are washed out. In practical applications one might not have unhindered control over flow rate and growth medium. In such a case eradication of the pathogen can be achieved by adding control agents to the system. Our analysis of the uncontrolled pathogenic system implies, however, that the overall longterm behavior as briefly described here is actually the result of the intricate interplay of self-inhibition, amensalism, and reactor conditions. In situations where nutrients become limited, these results might change because, depending on parameters, competition and amensalism may affect the dynamics between both species in opposite direction.

To the best of our knowledge this is the first study of this pH based amensalistic biopreservation mechanism. The model that we study here is somewhat idealized in the sense that it considers a completely mixed culture in the chemostat. While this is achievable in laboratory experiments, and while this is a reasonably good approximation of some bioreactors, many bacteria, including the pathogen *L. monocytogenes* that we consider in our study, in fact grow as bacterial biofilms, i.e., as spatially structured bacterial populations attached to a surface. While the biocontrol mechanism can be formulated in the biofilm context [31,32,33,34], such biofilm models presently cannot be upscaled to the reactor scale but only studied on the meso-scopic biofilm scale.

While out study was cast for *L. monocytogenes* and *L. lactis* and while we used functions of interaction and model parameters from the literaure specific to these two species, the qualitative results may apply to amensalistic systems more generally.

## Figures and Tables

**Table 1:** Reaction parameters used in this study, from [2].

Parameter	Symbol	Unit	*L. lactis*	*L. monocytogenes*
Specific growth rate	$\mu_{2},\mu_{1}$	h^−1^	0.1049	0.1471
Protonated acid production rate	δ,γ	Milimoles CFU^−1^·h^−1^	1.7 * 10^−10^	2.95 * 10^−10^
MIC acid (growth)	$k_{3},k_{7}$	Milimolar	5.2	4.058
MIC acid (metabolism)	$k_{4},k_{8}$	Milimolar	8.907	8.908
Maximum acid concentration	$k_{1},k_{2}$	Milimolar	11.5	11.65
MIC proton ion (growth)	$k_{5},k_{9}$	Milimolar	10^−1.405^	10^−1.892^
MIC proton ion (metabolism)	$k_{6},k_{10}$	Milimolar	10^−1.147^	10^−1.151^
Maximum proton ion concentration	$k_{11}$	Milimolar	10^−1.12^	10^−1.132^
Malate decay rate	*θ*	Milimole CFU^−1^·h^−1^	1.69 * 10^−10^	0
Malate utilization rate	*κ*	Milimole^−1^	10^−5.33^	10^−5.33^
proton concentration change rate	*ρ*	Moles CFU^−1^·h^−1^	10^−5.472^	10^−5.472^
